# Emerging Radiopharmaceuticals Beyond FDG for Ovarian Cancer: A Review of Advances in Nuclear Medicine

**DOI:** 10.1002/cam4.71167

**Published:** 2025-09-09

**Authors:** Mara M. K. Veenstra, Sophie Veldhuijzen van Zanten, Erik Vegt, Ingrid A. Boere, Heleen J. van Beekhuizen, Julie Nonnekens, Frederik A. Verburg, Maarten G. J. Thomeer

**Affiliations:** ^1^ Department of Radiology & Nuclear Medicine Erasmus MC – University Medical Centre Rotterdam Rotterdam the Netherlands; ^2^ Erasmus MC Cancer Institute Rotterdam the Netherlands; ^3^ Erasmus MC Transplant Institute Rotterdam the Netherlands; ^4^ Medical Delta Delft the Netherlands; ^5^ Department of Medical Oncology Erasmus MC – University Medical Centre Rotterdam Rotterdam the Netherlands; ^6^ Department of Gynaecological Oncology Erasmus MC – University Medical Centre Rotterdam Rotterdam the Netherlands; ^7^ Department of Molecular Genetics Erasmus MC – University Medical Centre Rotterdam Rotterdam the Netherlands

**Keywords:** diagnostic imaging, nuclear medicine, ovarian neoplasms, positron emission tomography, precision medicine, radionuclide imaging

## Abstract

**Aims:**

This review summarizes the role and future prospects of nuclear medicine in ovarian cancer, focusing on novel radiopharmaceuticals beyond FDG for diagnostic, predictive, and therapeutic applications within a theranostic framework.

**Materials and Methods:**

A narrative literature review was conducted using major databases. Peer‐reviewed articles addressing non‐FDG radiopharmaceuticals in ovarian cancer were identified and assessed; FDG‐based studies were excluded due to the availability of prior comprehensive reviews.

**Results:**

Novel radiopharmaceuticals show potential to enhance diagnostic accuracy, allow early evaluation of treatment response, predict chemotherapy resistance, and support stratification for targeted therapies. Several tracers are under investigation for theranostic use, offering combined diagnostic and therapeutic benefits.

**Discussion:**

Incorporating novel radiopharmaceuticals into ovarian cancer management may help overcome limitations of conventional imaging and systemic therapy. Theranostic strategies, uniting molecular imaging with radionuclide therapy, represent a promising step toward personalized medicine and could significantly influence clinical outcomes.

**Conclusion:**

Nuclear medicine, through innovative radiopharmaceuticals and theranostic approaches beyond FDG, is expected to expand its role in ovarian cancer care. Further research is needed to validate these applications and facilitate their integration into clinical practice.

## Graphical abstract

Emerging radiopharmaceuticals in nuclear medicine could improve ovarian cancer diagnosis and staging, while also enabling assessment of treatment response, therapy resistance, and suitability for targeted therapies.

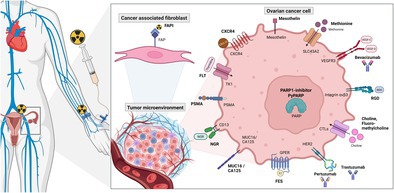

**Abbreviations**
BRCA: breast cancer gene
CA‐125: carbohydrate antigen‐125
CD13: aminopeptidase N
CT: computed tomography
CXCL12: C‐X‐C motif chemokine 12
CXCR4: C‐X‐C chemokine receptor 4
DWI: diffusion weight imaging
FAPI: fibroblast activation protein inhibitor
FDG: fluorodeoxyglucose
FES: fluoro‐17β‐oestradiol
FLT: fluorothymidine
HER2: human epidermal growth factor receptor 2
HSP90: heat shock protein 90
MRI: magnetic resonance imaging
mTOR: mammalian target of rapamycin
MUC16: mucin‐16
NGR: asparagine‐glycine‐arginine
PARP: poly (adenosine diphosphate‐ribose) polymerase
PET: positron emission tomography
PSMA: prostate specific membrane antigen
RGD: arginylglycylaspartic acid
SPECT: single‐photon emission computed tomography
SUV: standardised uptake value
VEGF: vascular endothelial growth factor

## 1 Introduction

Ovarian cancer is a heterogeneous and challenging disease, with high‐grade serous cancer being the most common type [1]. Most patients are diagnosed at an advanced stage and often experience recurrence despite treatment with cytoreductive surgery, platinum‐based chemotherapy, and maintenance therapies [2, 3]. Recurrence is commonly retreated with chemotherapy, sometimes combined with targeted therapies. However, platinum resistance remains a significant hurdle, [4] contributing to a poor prognosis with a 5‐year overall survival rate of just 45% [5].

Imaging is crucial for diagnosing, staging and managing ovarian cancer. Ultrasound and computed tomography (CT) are recommended in case of a suspicion of ovarian cancer [6]. Ultrasound, the primary diagnostic tool, is non‐invasive and inexpensive, but operator‐dependent [7]. CT is used for preoperative staging and postoperative monitoring [8]; but it struggles with differentiating malignant from benign (residual) lesions and detecting early stage cancer or small metastases [9, 10, 11]. For example, paracardiac lymph nodes can be hard to differentiate, but their disease status can distinguish between Stage III and Stage IV disease and therefore has treatment implications. Magnetic resonance imaging (MRI) outperforms CT in detecting peritoneal metastasis [12, 13, 14, 15], and diffusion‐weighted imaging can further improve lesion differentiation [16, 17, 18, 19, 20]. However, MRI is limited in its ability to differentiate lymph nodes based solely on diameter, requires advanced imaging sequences to minimise artefacts, and is highly reader dependent [21].

Nuclear medicine, particularly positron emission tomography (PET), has seen significant growth, although its use in ovarian cancer is not yet fully established. [^18^F]fluorodeoxyglucose (FDG) is the most widely used PET radiopharmaceutical, primarily used in ovarian cancer for detecting lymph node and distant metastases [22, 23, 24]. However, its accuracy can be limited by background glucose metabolism in the gastrointestinal tract and excretion via the urinary tract. Alternative radiopharmaceuticals may offer better solutions for assessing ovarian cancer.

**FIGURE 1:**
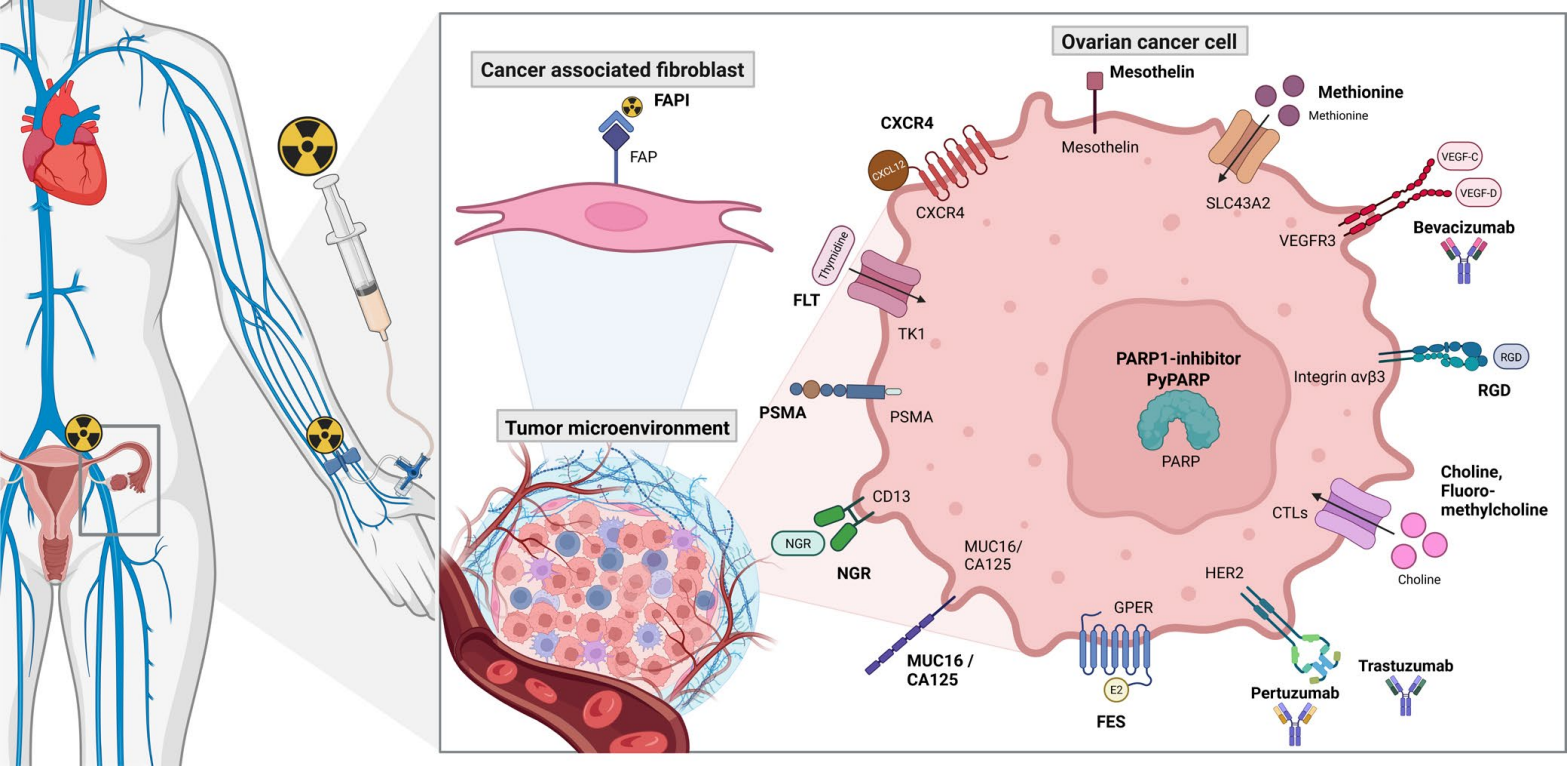
Schematic overview of the radiopharmaceuticals that are mentioned in this review. The radiopharmaceutical is administered through intravenous injection, where it enters the blood stream and attaches to its binding site: The binding site can be located on the cell surface, within the nucleus or on a different cell type from the tumour microenvironment. CA125, carbohydrate antigen 125; CD13, aminopeptidase N; CTLs, choline transporter‐like proteins; CXCL12, C‐X‐C motif chemokine 12; CXCR4, C‐X‐C chemokine receptor 4; E2, oestradiol; FAP(I), fibroblast activation protein (inhibitor); FES, fluoro‐17β‐oestradiol; FLT, fluorothymidine; GPER, G protein‐coupled oestrogen receptor; HER2, human epidermal growth factor receptor 2; MUC16, mucin 16; NGR, asparagine‐glycine‐arginine; PARP(i), Poly(adenosine diphosphate‐ribose) polymerase (inhibitor); PSMA, prostate specific membrane antigen; RGD, arginylglycylaspartic acid; SLC43A2, solute carrier family 43 member 2; TK1, thymidine kinase 1; VEGF(R), vascular endothelial growth factor (receptor). Created in BioRender. Veenstra (2025) https://BioRender.com/s16u284.

This review focuses on recent advancements in nuclear medicine for ovarian cancer, specifically non‐[^18^F]FDG radiopharmaceuticals (Figure 1) and explores their roles in diagnosis, staging, treatment selection, chemoresistance prediction, therapeutic monitoring, and their potential for radionuclide therapy.

## 2 Radiopharmaceuticals for Diagnosis and Staging of Ovarian Cancer

Over the last decade, several novel radiopharmaceuticals for diagnostic use in ovarian cancer have been studied.

### 2.1 Cell Proliferation Markers

**Fluorothymidine** (**FLT**), a thymidine (DNA precursor) analogue, can be used to image cell proliferation, which is, almost by definition, upregulated in malignancies. Apart from preclinical studies using ovarian cancer xenografts, [^18^F]FLT PET/CT has been tested in two studies that included women with ovarian cancer [25, 26]. Both studies involved three patients and found increased tumour uptake. Physiological [^18^F]FLT uptake is seen in bone marrow, liver, and the urinary tract, but not in the gastrointestinal tract like [^18^F]FDG [27]. A positive correlation between [^18^F]FLT uptake and Ki67 mitotic index was found [25], and [^18^F]FLT uptake correlated better with size reduction on CT after chemotherapy in three patients with recurrent ovarian cancer than [^18^F]FDG uptake [26].

**Methionine** is an essential amino acid for cell growth and division. [^11^C]methionine PET/CT for ovarian cancer has been studied in mice and patients (*n* = 13) [28, 29]. Tumours in mice showed high [^11^C]methionine uptake. In the patients, high [^11^C]methionine accumulation was observed in malignant tumours, while borderline malignant and benign lesions did not show any uptake. These studies, though small, implicate a possibility of using [^18^F]FLT and [^11^C]methionine as a diagnostic agent for ovarian cancer. Their performance compared with current standard imaging modalities, however, has yet to be evaluated.

**Choline**, a building block for cell membranes and thus essential for rapidly dividing cancer cells, is involved in several biological mechanisms and plays a role in cell membrane integrity [30]. Therefore, the increased proliferative activity in tumours can be visualised through radioactively labelled choline. Choline labelled with ^11^C or ^18^F has been used as a radiotracer for prostate cancer, and it is still used for localization of parathyroid adenomas. The use of [^11^C]choline PET compared with [^18^F]FDG PET in gynecologic cancers, mostly uterine (endometrial adenocarcinoma and carcinosarcoma), cervical (squamous cell), or ovarian cancer has previously been described in a cohort of 21 patients, of whom 18 had primary tumours (ovarian cancer *n* = 1) and three had recurrent ovarian cancer (total ovarian cancer *n* = 4) [31]. [^11^C]Choline PET was able to correctly identify more primary tumours than [^18^F]FDG PET (16/18 cases vs. 14/18 cases, respectively), but standardised uptake values (SUVs) were significantly higher for [^18^F]FDG. Both scans identified the primary tumour in the ovarian cancer patient. In the three patients with recurrent ovarian cancer, both scans were false‐negative in two and true‐positive in one of the patients. Also, [^11^C]choline PET showed physiologic bowel uptake that concealed para‐iliac lymph node metastases where [^18^F]FDG PET showed clear visibility, limiting its potential for ovarian cancer patients.

**Mesothelin** is a glycoprotein located at the cell surface that is believed to play a significant role in cell proliferation, growth, and adhesion signalling [32]. Mesothelin is overexpressed in many cancers and interacts with carbohydrate antigen‐125 (CA‐125, also known as mucin‐16 or MUC16). Normal tissue expression of mesothelin is limited to cells lining the peritoneum, pericardium, and pleura [33]. Mesothelin could potentially be used as an alternative to [^18^F]FDG to avoid uptake in the gastrointestinal tract, but small peritoneal and pleural metastases that frequently occur in ovarian cancer in particular may be missed, substantially reducing the usability of mesothelin. So far, ^64^Cu‐labelled mesothelin antibodies have been tested in mouse models of ovarian cancer and a total of four patients with ovarian cancer [34, 35]. Although initial in‐human results show promising visibility of ovarian cancer lesions mean SUV_max_ 14.5 (±8.7) and mean tumour‐to‐blood ratio 2.6 (±1.3), some peritoneal lesions were missed.

### 2.2 Angiogenesis Markers

Aminopeptidase N (also known as CD13) and integrin αvβ3 are two key regulators involved in tumour angiogenesis and tumour progression [36, 37]. Due to the varying expression levels of CD13 and integrin αvβ3 between ovarian cancer patients, their individual use as a target for radionuclide imaging is limited. To address this, Gai et al. [38] developed a dual‐receptor targeted radiopharmaceutical that consists of **asparagine‐glycine‐arginine (NGR)** and **arginylglycylaspartic acid** (**RGD**, the principal integrin‐binding domain in the extracellular matrix [39]). This tracer, called [^68^Ga]Ga‐NGR‐RGD, targets CD13 and integrin αvβ3. While this approach enhances the likelihood of tumour visualisation, it may not be effective in all patients, as some tumours do not overexpress either NGR or RGD. Long et al. [40] evaluated the effectiveness of [^68^Ga]Ga‐NGR‐RGD in ovarian cancer and found that [^68^Ga]Ga‐NGR‐RGD PET showed high tumour uptake in mouse models with subcutaneous xenografts. When compared with [^18^F]FDG PET, [^68^Ga]Ga‐NGR‐RGD PET also showed significantly higher tumour‐to‐muscle and tumour‐to‐liver ratios. [^68^Ga]Ga‐NGR‐RGD therefore provided better detail compared to [^18^F]FDG. In models of animals bearing abdominal metastasis, PET imaging with [^68^Ga]Ga‐NGR‐RGD enabled rapid and clear visualisation of both peritoneal and liver metastases (3–6 mm). In contrast, [^18^F]FDG failed to distinguish metastatic lesions due to the relatively low metabolic activity and higher physiological intestinal [^18^F]FDG uptake. The ability to detect small peritoneal metastases makes [^68^Ga]Ga‐NGR‐RGD PET an interesting tracer for further research in humans. If successful, [^68^Ga]Ga‐NGR‐RGD PET may allow for improved staging, improving clinical decision making.

**FIGURE 2:**
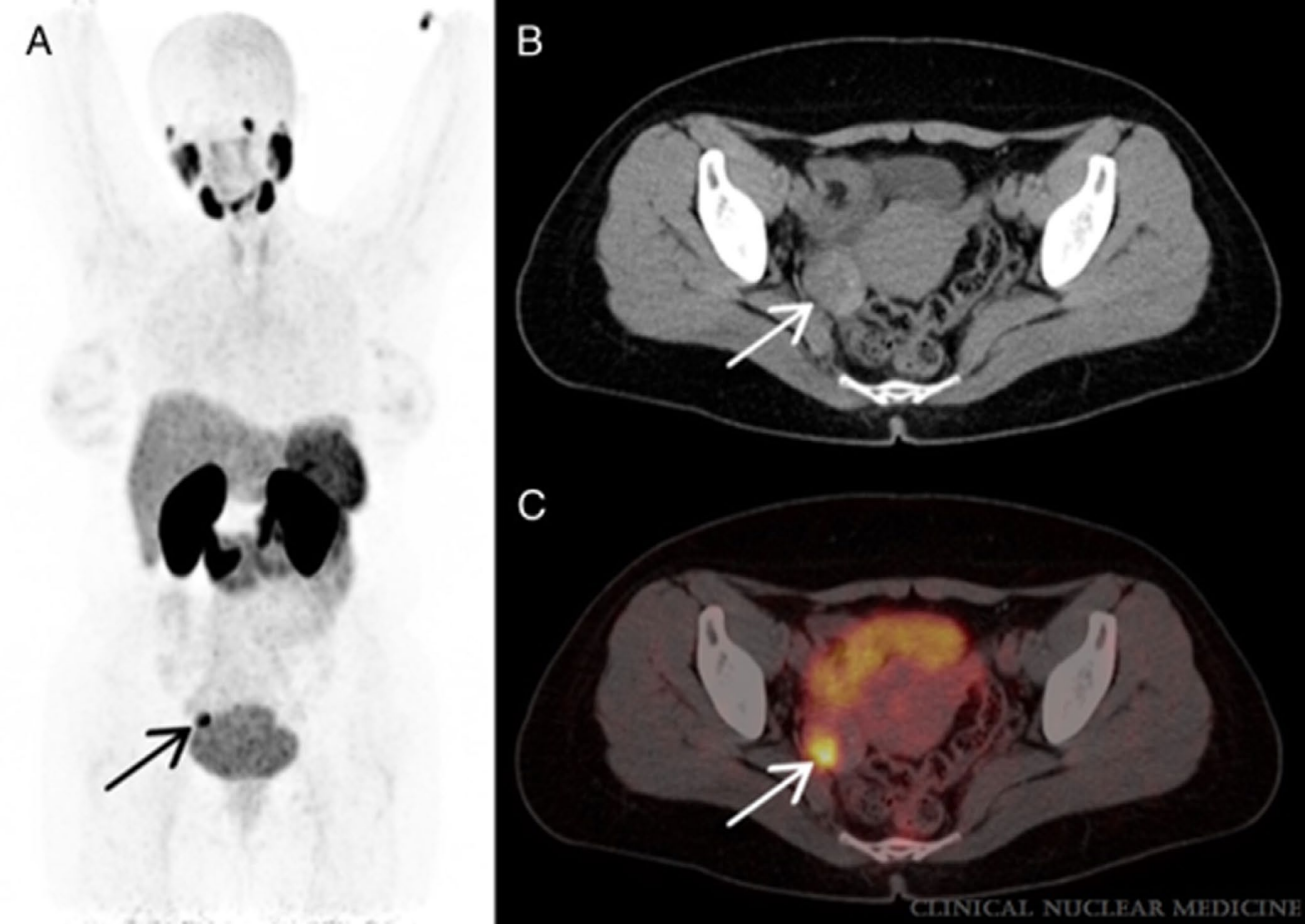
PET/CT scan with [^68^Ga]Ga‐PSMA‐11 in a 40‐year‐old woman. From Kunikowska et al. [44] (A) Maximum intensity projection, arrow indicates the lesion with abnormal tracer accumulation. (B) CT, arrow indicates the lesion in the right ovary. (C) Fusion PET/CT, arrow indicates the high tracer accumulation in the right ovary lesion visible on CT with SUV_max_ 13.8. Final histopathology revealed borderline ovarian tumour. Copyright 2022 Wolters Kluwer Health Inc. All rights reserved.

Another potential tumour vasculature target is the **prostate specific membrane antigen (PSMA)**, a type II membrane protein that is overexpressed in most prostate cancer cases and upregulated in the neovasculature of solid tumours [41, 42, 43]. Ligands targeting PSMA for locating ovarian cancer seem to be a feasible approach, as [^68^Ga]Ga‐PSMA‐11 showed increased tumour uptake on PET/CT, corresponding with contrast enhancement on diagnostic CT [44]. An example is shown in Figure 2. Similar results were found for [^18^F]DCFPyl PET/MRI, which also targets PSMA [45]. Metser et al. [46], however, found that [^18^F]DCFPyl PET/CT found fewer disease sites than CT, particularly in the upper abdomen and throughout the gastrointestinal tract, probably limiting its clinical utility. Whether or not [^68^Ga]Ga‐PSMA‐11 PET/CT has additional value over the currently used imaging modalities remains to be determined.

### 2.3 C‐X‐C Chemokine Receptor 4

**FIGURE 3:**
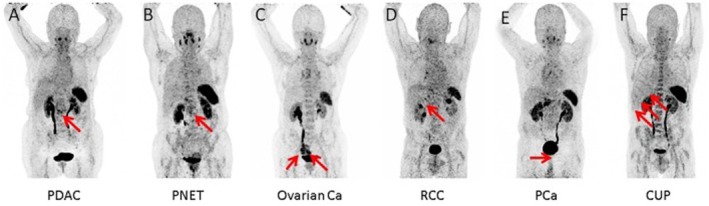
CXCR4‐directed positron emission tomography in several malignancies. From Werner et al. [51] Maximum intensity projections. The figure primarily demonstrates moderate to no uptake on CXCR4‐directed imaging (arrows), with the exception of the ovarian cancer patient, where the tumour masses display fairly high CXCR4 expression. (A) PDAC, pancreatic ductal adenocarcinoma; (B) PNET, pancreatic neuroendocrine tumour; (C) Ovarian cancer; (D) RCC, renal cell carcinoma; (E) PCa, prostate cancer. Copyright 2019 Werner, Kircher, Higuchi, Kircher, Schirbel, Wester, Buck, Pomper, Rowe and Lapa.

[^68^Ga]Ga‐CXCR4, a radiopharmaceutical for the **C‐X‐C chemokine receptor 4 (CXCR4)**, allows for sensitive and high‐contrast detection of the receptor's presence within the body [47, 48]. CXCR4 is expressed in primary ovarian tumours, [49] and [^68^Ga]Ga‐CXCR4 PET/CT has yielded positive results in studies investigating its use for the detection of ovarian cancer (Figure 3) [50, 51]. In these studies, a total of four patients underwent [^68^Ga]Ga‐CXCR4 PET/CT. Additional immunohistochemical analyses showed that tumours that were visible on [^68^Ga]Ga‐CXCR4 PET/CT (*n* = 2) had high CXCR4 expression, and tumours that were not (*n* = 2) had no or mild CXCR4 expression. Therefore, the diagnostic use of [^68^Ga]Ga‐CXCR4 PET/CT will probably be limited to tumours with high CXCR4 expression. However, Vag et al. [52] observed only low to moderate PET positivity using [^68^Ga]Ga‐CXCR4 in solid tumours that showed high CXCR4 expression in vitro. This discrepancy could indicate that transcript or whole‐cell protein level analysis results in different expression profiles than are seen using PET probes that bind to membrane‐associated chemokine receptors.

### 2.4 Fibroblast Activation Protein Inhibitor

**FIGURE 4:**
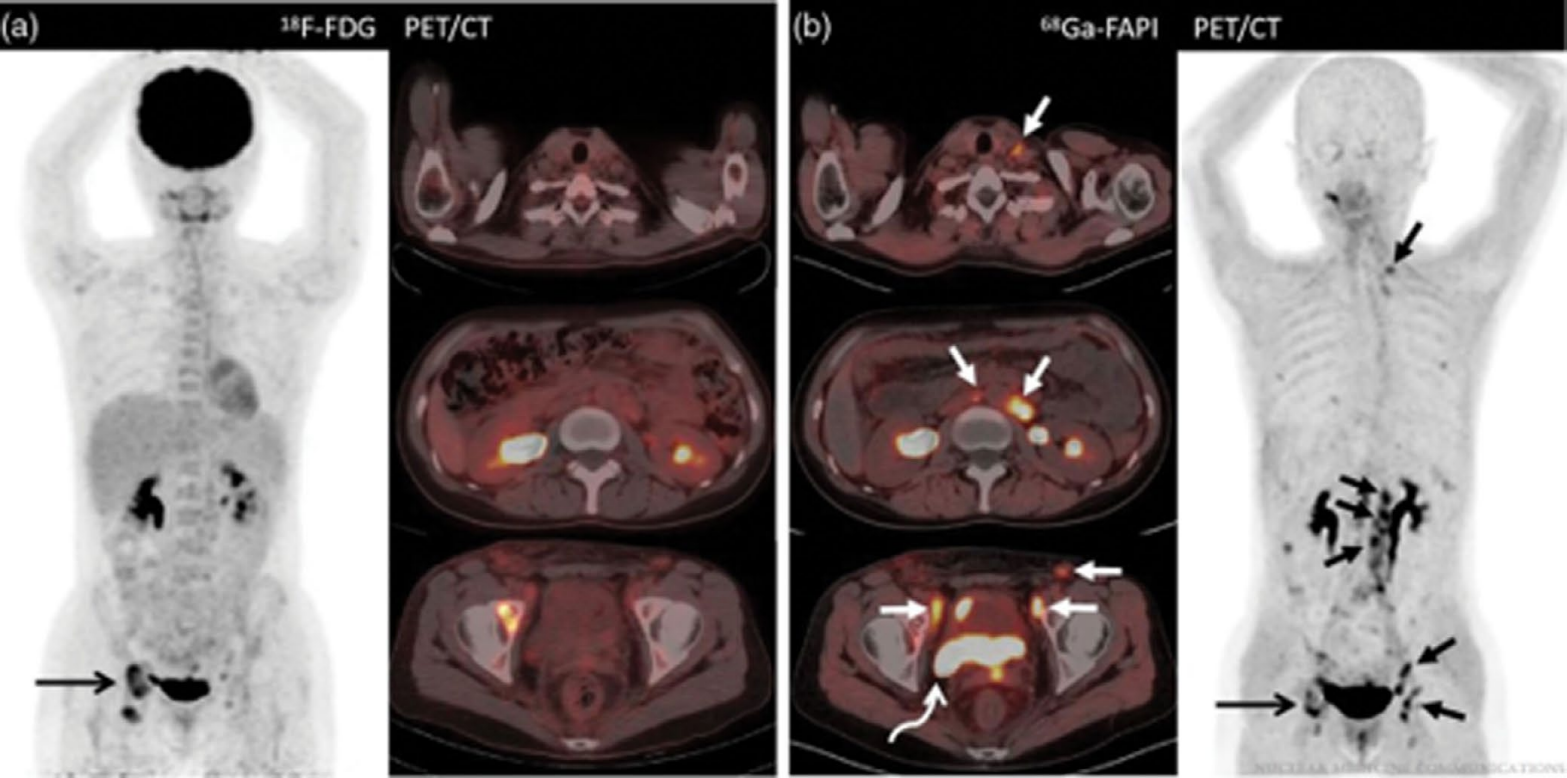
[^18^F]F‐FDG versus [^68^Ga]Ga‐FAPI‐04 positron emission tomography. From Zheng et al. [58] A 35‐year‐old woman who previously underwent surgery for poorly differentiated mucinous ovarian adenocarcinoma. Fluorine‐18‐fluorodeoxyglucose (^18^F‐FDG) positron emission tomography/computed tomography (PET/CT) (a) and gallium‐68‐labelled fibroblast activation protein inhibitor (FAPI)‐04 PET/CT (b) demonstrate intense uptake in the right ilium (long arrow). ^18^F‐FDG PET/CT showed no abnormal uptake throughout the body, whilst ^68^Ga‐FAPI PET/CT showed increased uptake in a cervical lymph node, the retroperitoneal lymph nodes, and the pelvic site of recurrence (bent arrow). Subsequent biopsy of the cervical node found metastatic ovarian serous carcinoma. Copyright 2022 The Author(s). Published by Wolters Kluwer Health Inc.

Perhaps one of the most promising radiopharmaceuticals for imaging is the **fibroblast activation protein** (FAP) **inhibitor (FAPI)**, which targets cancer‐associated fibroblasts that are present in the tumour microenvironment. FAP is proven to be absent in normal ovaries and most other organs, including the peritoneum, making it an appealing target for imaging of ovarian malignancies [53]. Several studies have compared ^68^Ga‐labelled FAPI with [^18^F]FDG PET in ovarian cancer patients (Figure 4). In all studies, FAPI PET outperformed [^18^F]FDG PET due to a higher sensitivity for diagnosing lymph node metastases [54, 55, 56, 57, 58, 59]. Furthermore, very low unspecific intestinal and peritoneal uptake was described [60]. One study compared ^68^Ga‐labelled FAPI PET/CT with MRI diffusion‐weighted imaging (DWI), also showing more favourable results for ^68^Ga‐labelled FAPI PET/CT [61]. FAPI PET/CT was especially better for detecting peritoneal tumour depositions and retroperitoneal, peri‐, or supradiaphragmatic lymph node metastases. In one study, 14% of treatment‐naïve and 33% of relapsed patients were upstaged following ^68^Ga‐labelled FAPI PET/CT compared with [^18^F]FDG PET/CT, resulting in treatment changes in 10% and 19% of cases, respectively, highlighting the clinical impact of FAPI PET/CT in ovarian cancer [54].

Through recent years, several radiopharmaceuticals have been studied for their diagnostic use in ovarian cancer. The current development status of the radiopharmaceuticals mentioned in this review is shown in Table 1, and a short comparative overview can be found in Table 2. Choline, mainly used to study parathyroid disease, and PSMA, to diagnose and monitor prostate cancer, are already routinely used in clinical practice, possibly increasing their chances of being implemented in ovarian cancer diagnostics. Limitations of these radiopharmaceuticals, however, include physiological uptake within the gastrointestinal tract that could cause metastases to be missed, limiting sensitivity. Therefore, we suggest that FAPI seems to be the most promising tracer for future clinical implementation in ovarian cancer. FAPI is increasingly being studied for several cancer types in Phase 2 clinical trials (e.g., NCT05209750, NCT06355427, NCT05263700, NCT05898854, NCT05262855) and has been integrated into clinical practice in a few settings. Although FAPI PET/CT shows considerable diagnostic promise, its cost‐effectiveness has not yet been formally evaluated, highlighting the need for future health economic studies to assess its value relative to other established imaging modalities. ^68^Ga‐labelled FAPI for ovarian cancer is currently being investigated in a few clinical trials (NCT06232122, NCT05903807, NCT06061874) with results expected around 2026.

**TABLE 1:** Current development status of radiopharmaceuticals used in ovarian cancer.

Radiopharmaceutical	Research phase in ovarian cancer	Research phase in other diseases	Current clinical trials in ovarian cancer^a^
[^89^Zr]Bevacizumab	Preclinical	Phase 2	—
[^11^C]Choline	Phase 1	Phase 4	—
[^177^Lu]Lu‐CHX‐A″‐DTPA‐huAR9.6	Preclinical	Preclinical	—
[^64^Cu]Cu‐cyclam‐RAFT‐c(‐RGDfK‐)_4_	Preclinical	Preclinical	—
[^64^Cu]Cu‐DOTA‐trastuzumab	Preclinical	Phase 1	—
[^18^F]DCFPyL	Phase 1	Phase 4	—
[^68^Ga]Ga‐DOTA‐FAPI‐04	Phase 1	Phase 1	NCT05824247
[^68^Ga]Ga ‐FAPI‐02	Phase 1	Phase 1	—
[^68^Ga]Ga ‐FAPI‐04	Phase 1	Phase 2	—
[^68^Ga]Ga ‐FAPI‐46	Phase 1	Phase 2	NCT06232122 NCT05903807 NCT06061874 NCT05172310 NCT04147494
[^18^F]FAPI‐74	Phase 2	Phase 2	NCT06503146
[^68^Ga]Ga‐PNT6555 (FAPI)	Phase 1	Phase 1	NCT05956093
[^18^F]FES	Phase 2	Phase 4	—
[^18^F]FLT	Phase 1	Phase 2	—
[^18^F]Fluoromethylcholine	Preclinical	Phase 3	—
[^18^F]FPPRGD_2_	Phase 1	Phase 1	—
[^18^F]PARP1‐inhibitor	Phase 1	Phase 2	NCT02637934 NCT03604315
[^64^Cu]Cu‐Mesothelin	Preclinical	Preclinical	—
[^89^Zr]Mesothelin	Phase 1	Phase 1	—
[^11^C]Methionine	Phase 1	Phase 2	—
[^68^Ga]Ga‐NGR‐RGD	Preclinical	Preclinical	—
[^64^Cu]Cu‐NOTA‐pertuzumab F(ab')2	Preclinical	Preclinical	—
[^68^Ga]Ga‐CXCR4	Phase 1	Phase 2	—
[^177^Lu]Lu‐CXCR4	—	Phase 1	
[^90^Y]Y‐CXCR4	—	Phase 1	
[^68^Ga]Ga‐PSMA‐11	Phase 1	Phase 4	NCT04147494
[^18^F]PyPARP	Preclinical	Phase 1	—
[^89^Zr]Trastuzumab	Preclinical	Phase 2	—

*Note:* Overview of the radiopharmaceuticals that are explored in this review, including information on their current research phase.

^a^ Reviewed on ClinicalTrials.gov on January 28th 2025.

**TABLE 2:** Key diagnostic and functional properties of radiopharmaceuticals studied in ovarian cancer.

Radiopharmaceutical	Molecular target	Theranostic application	Key strengths	Key limitations
[^89^Zr]Bevacizumab	Vascular endothelial growth factor A	Potential	High specificity for angiogenesis imaging	Long half‐life of ^89^Zr; limited availability
[^11^C]Choline [^18^F]Fluoromethylcholine	Cell membrane synthesis	Potential	Evaluates tumour metabolism	Short half‐life of ^11^C requires on‐site cyclotron; limited data in ovarian cancer
[^68^Ga]Ga‐CXCR4	C‐X‐C Chemokine Receptor 4 (CXCR4)	Yes	Potential for both imaging and therapy	CXCR4 expression may vary; limited clinical experience
[^18^F]FAPI‐74 [^68^Ga]Ga‐FAPI‐46	Fibroblast activation protein	Yes	High tumour‐to‐background ratio; ^18^F‐label allows centralised production; early clinical use	Limited outcome data in ovarian cancer; still in early phase trials
[^18^F]FES	Oestrogen receptor (ER)	Potential	Clinically validated in breast cancer; ER‐targeted	Only applicable to ER+ tumours, limited ovarian use
[^18^F]FLT	Thymidine kinase‐1	Potential	Non‐invasive marker of cellular proliferation	Uptake influenced by factors beyond proliferation; limited therapeutic translation so far
[^18^F]FPPRGD_2_	Integrin αvβ3	Potential	Visualises tumour angiogenesis and integrin expression	Limited availability; early phase clinical data only
[^89^Zr]Mesothelin	Mesothelin	Potential	Tumour‐specific antigen with therapeutic potential	Variable expression in ovarian cancer; early stage development
[^11^C]Methionine	Amino acid transport	Potential	Evaluates amino acid metabolism	Short half‐life of ^11^C; limited widespread use
[^18^F]PARP1‐inhibitor [^18^F]PyPARP	Poly(Adenosine diphosphate‐Ribose)Polymerase (PARP)	Potential	May inform PARP‐inhibitor therapy; DNA repair marker	Still investigational; limited clinical experience
[^68^Ga]Ga‐PSMA‐11 [^18^F]DCFPyL	Prostate specific membrane antigen	Yes	Theranostic potential; well established in prostate cancer	Limited expression in ovarian tumours
[^89^Zr]Trastuzumab [^64^Cu]Cu‐DOTA‐trastuzumab	Human Epidermal growth factor Receptor 2 (HER2)	Potential	Targets HER2‐overexpressing tumours	Limited to HER2+ cases; long half‐life of ^89^Zr

*Note:* Overview of clinically tested radiopharmaceuticals that are investigated in this review, highlighting their molecular targets, potential theranostic applications, and key strengths and limitations.

## 3 Patient Selection for Targeted Therapy

The previously mentioned radiopharmaceuticals may theoretically also be used to select patients for treatment. The following paragraphs review the current radiopharmaceutical developments in this regard.

### 3.1 Poly‐ADP‐Ribonuclease Inhibitors

**FIGURE 5:**
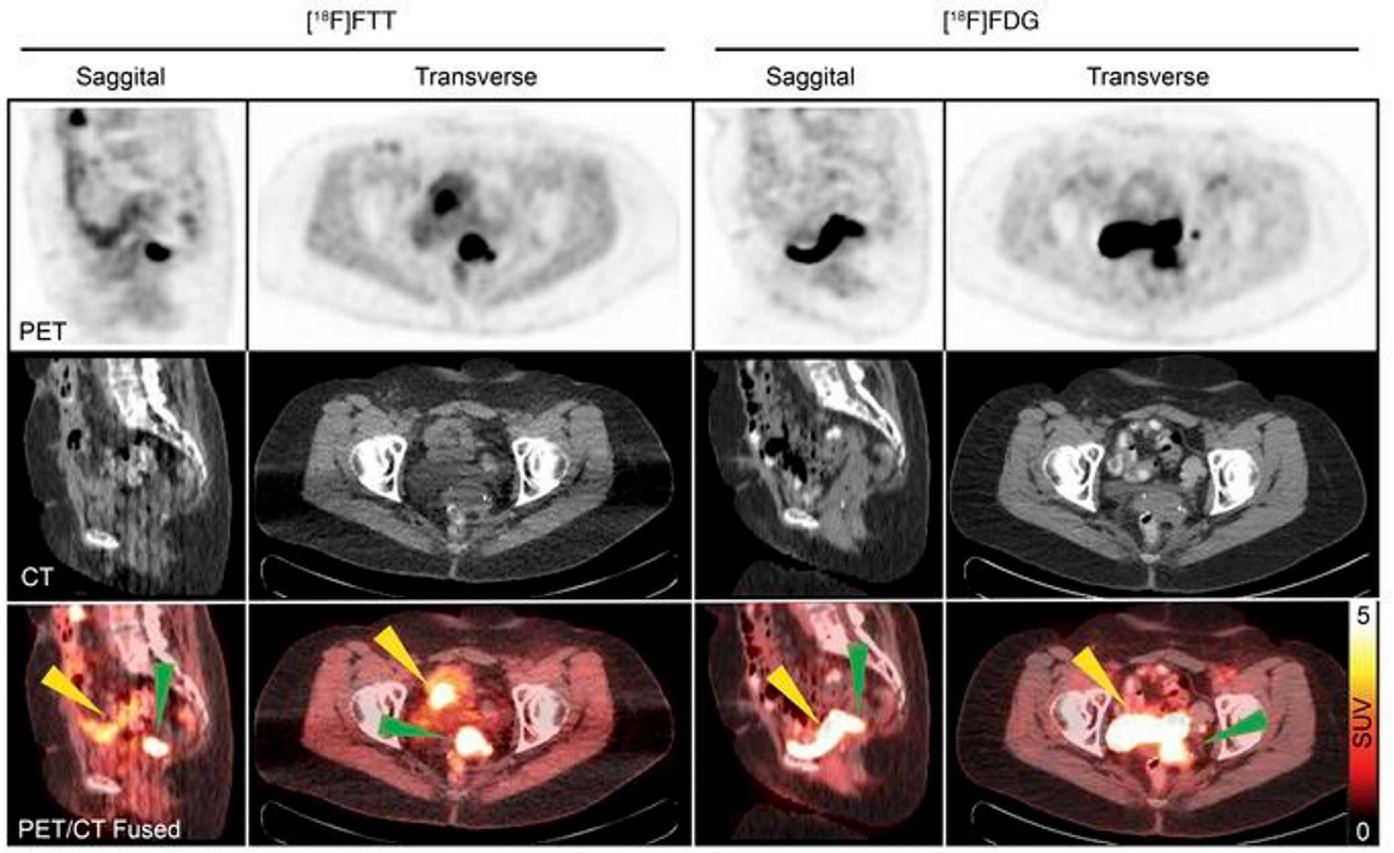
[^18^F]PARP1‐inhibitor and [^18^F]FDG PET/CT images of a patient with ovarian cancer with vaginal cuff lesion. From Makvandi et al. [65] [^18^F]FTT (left) is a [^18^F]PARP1‐inhibitor. Minimal radiotracer in the urinary bladder with [^18^F]PARP1‐inhibitor PET allowed for clear visualisation of the lesion (green arrow) with no interference, despite some bowel uptake (yellow arrow on [^18^F]PARP1‐inhibitor image). Note excreted radiotracer in the bladder on [^18^F]FDG PET (yellow arrow on [^18^F]FDG PET). Copyright 2018, American Society for Clinical Investigation.

One of the largest advances in the treatment of ovarian cancer is the use of poly(adenosine diphosphate‐ribose)polymerase (PARP) inhibitors. PARP enzymes attach to DNA breaks and attract other DNA damage repair proteins to repair the DNA [62]. PARP‐inhibitors block the enzyme on single‐strand DNA breaks, preventing the repair of DNA damage through poly(ADP‐ribosyl)ation (parylation), leading to the accumulation of DNA breaks and tumour cell death. This treatment is most effective in tumours with deficiencies in homologous recombination repair, which is responsible for repairing double‐strand breaks in the DNA. The PARP1 enzyme can be visualised using radiolabelled ligands, of which a **fluorine‐labelled PARP1 inhibitor** ([^18^F]PARP1‐inhibitor) currently seems the most promising [63, 64]. Interim analyses of an ongoing clinical trial (NCT02637934) evaluating [^18^F]PARP1‐inhibitor PET in ovarian cancer compared results of [^18^F]PARP1‐inhibitor PET with [^18^F]FDG PET in eight and 14 patients, respectively [65, 66]. [^18^F]PARP1‐inhibitor PET showed limited bladder activity because of biliary excretion and showed better identification of primary and recurrent lesions within the pelvis (Figure 5). Contrarily, [^18^F]FDG was better at detecting peritoneal metastasis. The authors found that high [^18^F]PARP1‐inhibitor uptake following carboplatin and paclitaxel treatment was indicative of chemoresistance, while low [^18^F]PARP1‐inhibitor uptake following the same therapy was indicative of chemosensitivity [65]. These results imply that [^18^F]PARP1‐inhibitor PET can be used to guide clinical management in combination with currently used biomarkers. Furthermore, differences in uptake were found within the tumour itself, suggesting that [^18^F]PARP1‐inhibitor PET may be able to visualise differences in PARP1 expression within a tumour and between different metastases in the same patient. In addition to this advantage over invasive biopsies, PARP inhibitor PET would also enable repeated imaging to determine (loss of) PARP1 expression during therapy. Interestingly, the authors described high uptake of [^18^F]PARP1‐inhibitor in five low‐grade ovarian cancer lesions [66]. If confirmed, [^18^F]PARP1‐inhibitor PET may serve as a biomarker to identify low‐grade ovarian cancer patients with unexpectedly high PARP1 expression, indicating potential sensitivity to PARP inhibitors, even if they do not have breast cancer gene (BRCA) mutations or homologous recombination deficiency. A disadvantage of [^18^F]PARP1‐inhibitor PET, however, would be its hepatobiliary excretion that could interfere with the detection of abdominal lesions. A novel PARP ligand, [^18^F]**PyPARP**, shows a much lower liver‐to‐kidney ratio, which could diminish the interference of hepatobiliary excretion [67]. On the other hand, the increased renal excretion may obscure pelvic lesions.

### 3.2 Anti‐Hormonal Treatment

A relatively less frequent subtype of ovarian cancer, low‐grade serous ovarian cancer, is often positive for the oestrogen and progesterone receptor, and whereas these tumours are relatively resistant to chemotherapy, they may respond to hormonal therapy [68, 69, 70]. Van Kruchten et al. [71] performed [^18^F]**fluoro‐17β‐oestradiol (FES)** PET/CT, that visualises the oestrogen receptor, in 15 suspected ovarian cancer patients that were planned to undergo surgery. A total of 28/32 (88%) lesions larger than 10 mm that were present on CT were quantified using [^18^F]FES PET/CT. The other four lesions were visible on PET, but not quantifiable due to high uptake levels in adjacent tissues. There were no new lesions that had not yet been found on diagnostic CT. This study also showed that [^18^F]FES PET/CT was consistent with histology at cytoreductive surgery [71]. These results indicate that [^18^F]FES PET/CT could potentially be used to indicate the current oestrogen receptor status, potentially identifying patients with high oestrogen receptor expression levels that could benefit from anti‐hormonal therapy.

### 3.3 Anti‐HER2 Treatment

Human epidermal growth factor receptor 2 (HER2) expression and/or gene amplification can be found in a subset of ovarian cancer (in up to 30%–40%), depending on the cutoff for receptor positivity [72, 73, 74]. Hence, anti‐HER2 therapies such as trastuzumab, pertuzumab, and tyrosine kinase inhibitors have been tested for HER2‐positive ovarian cancer. Newer agents also include antibody‐drug conjugates targeting the HER2 receptor, such as trastuzumab deruxtecan, for which response rates of up to 45% have been reported in ovarian cancer patients with HER2‐positive disease (HER2 3+ or amplification) [75]. Efficacy has also been observed in ovarian cancers with lower HER2 expression (20% response in HER2 1+) [75]. Radiopharmaceuticals can be used to non‐invasively monitor the expression of HER2, which is involved in tumour cell proliferation and metastasis. In preclinical ovarian cancer models, **trastuzumab**‐ and **pertuzumab**‐based imaging have demonstrated a decrease in HER2 expression following trastuzumab or heat shock protein 90 (HSP90)‐targeted therapy, providing a measurable response [76, 77, 78]. Performing a baseline HER2‐targeted PET/CT might therefore be able to detect patients with high HER2 expression levels, suggesting they could benefit from HER2‐directed treatments. The extent to which radiolabelled HER2 antibodies will be of clinical use, however, is still unknown.

### 3.4 Chemotherapy

**C‐X‐C chemokine receptor 4** (CXCR4) functions by binding to its ligand, the C‐X‐C motif chemokine 12 (CXCL12), causing changes in cell skeleton rearrangement and prompting cell migration [79]. The activation of CXCR4 and the migration of cancer cells towards organs that express CXCL12 facilitate the directed metastasis of cancer cells [80, 81, 82]. Studies have found that interfering with CXCR4 expression or blocking the CXCR4‐CXCL12 axis using small interfering RNA (siRNA) or other inhibitors (i.e., Plerixafor, TN14003, AMD3100) significantly reduces cell viability, invasion, migration, and adhesion of cancer cells in vitro [83, 84, 85, 86, 87]. [^68^Ga]Ga‐CXCR4 PET/CT may therefore enable clinicians to identify patients who could benefit from CXCR4‐directed drugs, although these drugs are not yet available clinically. The CXCR4‐CXCL12 pathway could also be used to predict chemosensitivity. Literature has shown high CXCR4 levels to be associated with resistance to platinum‐based chemotherapy [85, 88, 89]. Visualisation of increased CXCR4 expression using [^68^Ga]Ga‐CXCR4 PET/CT might therefore be able to show a correlation between the level of CXCR4 and a patient's likelihood to be resistant to platinum‐based chemotherapy, which would be of great benefit for clinical decision making.

Similar prospects for the prediction of chemosensitivity have been described for [^11^C]**choline** and [^18^F]**fluoromethylcholine**. In a study by Jimbo et al. [90], metastatic castrate‐resistant prostate cancer patients with a ≥ 20% reduction in SUV_max_ as measured on [^11^C]Choline PET/CT following three cycles of docetaxel chemotherapy, compared with their baseline SUV_max_ prior to starting docetaxel, were 3.6 times more likely to have a complete response after six full cycles of docetaxel chemotherapy. Choline uptake and its relation to chemosensitivity have not yet been studied in patients with ovarian cancer, however. Bauerschlag et al. [91] reported that cisplatin‐resistant cells displayed lower [^18^F]fluoromethylcholine uptake than cisplatin‐sensitive cells, which is why baseline imaging using this ligand might also be able to predict which patients could benefit from cisplatin. So far, this method has not yet been translated to clinical use. The high prevalence of chemoresistance in ovarian cancer patients and the burden of undergoing systemic treatment, however, do provide a clinical incentive to further assess the full potential of these ligands.

## 4 Monitoring Response to Treatment

Radiopharmaceuticals that are capable of diagnosing cancer are often also useful to assess therapy response. Few studies on novel radiopharmaceuticals have investigated (early) response to ovarian cancer treatment; they are briefly presented in the following paragraphs.

### 4.1 Chemotherapy and/or Targeted Therapy

Evidence has been presented that [^18^F]**fluorothymidine** ([^18^F]FLT) PET can assess treatment efficacy following carboplatin, paclitaxel, belinostat, and nicotinamide phosphoribosyltransferase inhibitors (APO866) [92, 93, 94]. [^18^F]FLT PET has also been used to monitor early treatment response in cisplatin‐resistant ovarian tumours and those treated with mammalian target of rapamycin (mTOR) inhibitors [28, 95, 96]. Additionally, one clinical study that assessed gemcitabine‐based secondary systemic treatment in three women with recurrent ovarian cancer suggested that [^18^F]FLT PET may become the new monitoring standard for this indication, as [^18^F]FLT PET detected treatment response quicker than [^18^F]FDG PET and showed a decrease in standardised uptake values that was better correlated with CT than [^18^F]FDG PET [26]. Similar findings were found for the use of [^11^C]**methionine** in a study with mice showing a significant decrease in tumour size and lesion uptake following chemotherapy [28]. More extensive research is needed to confirm these findings.

### 4.2 Anti‐Angiogenic Therapy

Other studies assessed the use of the vascular endothelial growth factor (VEGF). VEGF plays an important role in tumour angiogenesis [97]. It can be targeted through the humanised monoclonal antibody **bevacizumab**, and can be visualised using the radioactively labelled [^89^Zr]bevacizumab. Studies using ovarian cancer mouse models have found that [^89^Zr]bevacizumab was successfully able to monitor treatment response following HSP90 and mTOR inhibitors [98, 99]. [^89^Zr]bevacizumab uptake decreased by 44% following 2 weeks of twice‐weekly HSP90‐inhibition therapy and by 22% following daily mTOR‐inhibition for 2 weeks. Simultaneously, tumours in the treatment groups showed significantly slower tumour growth compared with the control groups. It would be interesting to see if radiolabelled‐bevacizumab PET is effective in monitoring early treatment response in ovarian cancer patients.

A study that used radiolabelled **arginylglycylaspartic acid** (RGD) analogues also showed promising results for monitoring response to therapy. Minamimoto et al. [100] used [^18^F]FPPRGD_2_ PET/CT in a total of six patients suffering from ovarian or cervical cancer. Patients were treated with bevacizumab‐containing therapy and monitored by [^18^F]FPPRGD_2_ PET/CT scans. Results showed that uptake decreased significantly during the course of therapy, suggesting that [^18^F]FPPRGD_2_ has the potential to provide early predictions of response to bevacizumab‐containing treatments.

## 5 Radiopharmaceuticals as Treatment

Radiopharmaceuticals can also serve as therapeutic agents for delivering internal radiation, a strategy known as targeted radionuclide therapy. When radiopharmaceuticals are used for both diagnostic and therapeutic purposes, they are referred to as *theranostic* pairs.

### 5.1 C‐X‐C Chemokine Receptor 4

C‐X‐C chemokine receptor 4 (CXCR4) is a theranostic agent that can be labelled with gallium‐68 for diagnostic purposes and lutetium‐177 or yttrium‐90 for treatment purposes. So far, [^177^Lu]Lu−/[^90^Y]Y‐CXCR4 has been used in patients suffering from several types of blood cancers [101, 102, 103, 104]. Their application served as neoadjuvant treatment before undergoing allogenic or autologous haematopoietic stem cell transplantation. Although [^177^Lu]Lu−/[^90^Y]Y‐CXCR4 will require stem cell replacement because of bone marrow ablation, this CXCR4‐targeted therapy could potentially also be administered to patients with solid tumours, such as end‐stage ovarian cancer patients, that show increased CXCR4 expression on PET [50, 105].

### 5.2 Fibroblast Activation Protein Inhibitor

A suitable alternative for future theranostic applications could be radiolabelled fibroblast activation protein inhibitor (FAPI), as was recently concluded in a review by Privé and colleagues [106]. FAP‐targeted radionuclide therapy performed on a compassionate use basis has shown effectiveness in several malignancies, but response in ovarian cancer patients has not yet been analysed. So far, results show a favourable toxicity profile with limited high‐grade adverse events, which were manageable if present [106]. The most relevant side effects are related to bone marrow toxicity, such as anaemia, leukopenia, and low platelets. However, a limitation of FAPI‐based therapies is the short tumour retention of current FAPI tracers. This fast clearance from the tumour reduces the radiation dose that radiolabelled FAPI delivers to a tumour, limiting its therapeutic efficacy.

### 5.3 Prostate Specific Membrane Antigen

Fast clearance could also pose challenges for the use of prostate specific membrane antigen (PSMA) radioligand therapy. PSMA is often only expressed on neovasculature in non‐prostate cancer, which is why uptake on endothelial cells results in a rapid tumour washout [107, 108, 109]. ^177^Lu‐labelled PSMA therapy has, however, been studied extensively in prostate cancer patients and shows effectiveness with a well manageable toxicity profile [110, 111]. Like FAPI, PSMA targeted radioligand therapy is yet to be researched in ovarian cancer patients. The favourable safety profile and promising results for these therapies encourage further investigation into their use for several malignancies, including ovarian cancer.

### 5.4 Arginylglycylaspartic Acid and Carbohydrate Antigen‐125

Some other radiopharmaceuticals have been described for their potential therapeutic use in ovarian cancer. These include radiolabelled arginylglycylaspartic acid (RGD) analogues for the management of peritoneal metastases [112] and use of carbohydrate antigen‐125 (MUC16/CA‐125), which was analysed in a biodistribution study [113] [^64^Cu]Cu‐cyclam‐RAFT‐c(‐RGDfK‐)_4_ PET was able to adequately visualise peritoneal metastases in mice. A therapeutic dose resulted in decreased tumour cell proliferation and increased apoptosis. [^177^Lu]Lu‐CHX‐A″‐DTPAhuAR9.6, that targets MUC16, showed a strong cytotoxic effect in tumours with high MUC16‐expression levels. Mice treated with this agent survived significantly longer than mice treated with saline. Both theranostic agents showed negligible toxicity. Whether or not these treatments are suitable for clinical use in humans remains to be further explored.

## 6 Current Challenges: Translating Research Into Practice

Over the last years, exciting discoveries have been made within the field of nuclear medicine, especially using cancer cell lines and mouse models. However, many promising targets remain to be assessed in patients. Clinical results so far mainly originate from *compassionate use*. This implies that access to this therapy remains limited for certain populations globally, since guidelines regarding *compassionate use* vary per country. Prospective clinical trials are therefore urgently needed to translate promising research results to patients. The ability to translate novel radiopharmaceuticals into clinical care does not only depend on scientific progress, but also on the healthcare system in which they are introduced. Access to cyclotrons and radiopharmaceuticals varies greatly across healthcare settings. While well‐funded academic institutions may operate on‐site cyclotrons or collaborate with regional radiopharmacies, smaller or resource‐limited hospitals typically rely on commercially available or generator‐based agents. New radiopharmaceuticals often require extensive documentation, including toxicity, dosimetry, and clinical data, often calling for dedicated research staff to help guide regulatory submissions and ethics approval. Furthermore, for promising radiopharmaceutical therapies to be used in patients, hospitals need proper equipment such as a cyclotron or generator, secured facilities with up‐to‐date certifications to safely work with radioactive materials, and ample funds to perform this kind of research and clinical care. Alternatively, radiopharmaceuticals could be provided by external companies that specialise in this area. Moreover, due to the short half‐life of some radiopharmaceuticals used in patients, research and clinical facilities using these drugs must be located near the manufacturing companies. Consequently, translational research in nuclear medicine remains complex. The lack of in‐human results following promising preclinical findings does not necessarily indicate that a radiopharmaceutical is unsuitable for clinical application. Nevertheless, the rapid expansion of nuclear medicine highlights its integration into modern medicine, offering promising potential for future translations.

## 7 Conclusions

Several radiopharmaceuticals have been studied for their potential use in ovarian cancer through recent years. New radiopharmaceuticals such as radiolabelled FAPI may be of great diagnostic use for ovarian cancer by detecting small metastases or distinguishing between benign and malignant lesions. Even though the number of studied cases remains small, PET also has the prospect of successful use in monitoring disease (e.g., PARP‐targeted PET), monitoring therapeutic effects (e.g., FLT PET), predicting which patients are prone to therapy resistance (e.g., CXCR4 PET), and non‐invasively identifying patients who could benefit from novel pharmacological treatments (e.g., FES PET), while targeted radionuclide therapy has high potential for future clinical treatment in ovarian cancer.

## Author Contributions

**Mara M. K. Veenstra:** investigation (lead), writing – original draft (lead). **Sophie Veldhuijzen van Zanten:** writing – review and editing (equal). **Erik Vegt:** writing – review and editing (equal). **Ingrid A. Boere:** writing – review and editing (equal). **Heleen J. van Beekhuizen:** writing – review and editing (equal). **Julie Nonnekens:** writing – review and editing (equal). **Frederik A. Verburg:** supervision (supporting), writing – review and editing (equal). **Maarten G. J. Thomeer:** conceptualization (lead), supervision (lead), writing – review and editing (equal).

## Conflicts of Interest

The authors declare no conflicts of interest.

## Data Availability

Data sharing is not applicable to this article as no new data were generated or analysed.
